# Enhanced Archaerhodopsin Fluorescent Protein Voltage Indicators

**DOI:** 10.1371/journal.pone.0066959

**Published:** 2013-06-19

**Authors:** Yiyang Gong, Jin Zhong Li, Mark J. Schnitzer

**Affiliations:** 1 James H. Clark Center, Stanford University, Stanford, California, United States of America; 2 CNC Program, Stanford University, Stanford, California, United States of America; 3 Howard Hughes Medical Institute, Stanford University, Stanford, California, United States of America; Institut Curie, France

## Abstract

A longstanding goal in neuroscience has been to develop techniques for imaging the voltage dynamics of genetically defined subsets of neurons. Optical sensors of transmembrane voltage would enhance studies of neural activity in contexts ranging from individual neurons cultured *in vitro* to neuronal populations in awake-behaving animals. Recent progress has identified Archaerhodopsin (Arch) based sensors as a promising, genetically encoded class of fluorescent voltage indicators that can report single action potentials. Wild-type Arch exhibits sub-millisecond fluorescence responses to trans-membrane voltage, but its light-activated proton pump also responds to the imaging illumination. An Arch mutant (Arch-D95N) exhibits no photocurrent, but has a slower, ~40 ms response to voltage transients. Here we present Arch-derived voltage sensors with trafficking signals that enhance their localization to the neural membrane. We also describe Arch mutant sensors (Arch-EEN and -EEQ) that exhibit faster kinetics and greater fluorescence dynamic range than Arch-D95N, and no photocurrent at the illumination intensities normally used for imaging. We benchmarked these voltage sensors regarding their spike detection fidelity by using a signal detection theoretic framework that takes into account the experimentally measured photon shot noise and optical waveforms for single action potentials. This analysis revealed that by combining the sequence mutations and enhanced trafficking sequences, the new sensors improved the fidelity of spike detection by nearly three-fold in comparison to Arch-D95N.

## Introduction

Fluorescent protein indicators of trans-membrane voltage offer the prospect of directly imaging the voltage dynamics or spiking activity of genetically defined cell types [1,2]. For example, optical experiments using such sensors might probe the biophysical dynamics of individual neurons, or the network dynamics of neuronal ensembles. Over prior decades, there have been many optical studies that used voltage-sensitive dyes to record action potentials or synaptic potentials in individual neurons or neuronal populations [3–5]. Small molecule optical voltage sensors can exhibit substantial signals (Δ*F*/*F* > 10%) and microsecond kinetics, and have thus revealed neuronal dynamics across a variety of settings [5,6]. Today, there is a rapidly growing set of genetic tools for dissecting neural circuit function, and a genetically encoded voltage indicator would be a key addition to this toolkit. Moreover, continual long-term expression of the sensor protein would open up the possibility of time-lapse experiments over multiple days or weeks. There are hybrid approaches that combine the expression of a genetically encoded fluorescent protein with the addition of an exogenous small molecule, but these require the application of the exogenous agent prior to each imaging session and have not been used for long-term imaging [7,8]. Genetically encoded fluorescent indicators of intracellular [Ca^2+^] already have a major role in neuroscience research, but in many neuron types Ca^2+^ imaging poorly captures spiking dynamics. Moreover, Ca^2+^ imaging generally does not provide the capability to examine sub-threshold voltage dynamics or high frequency spiking. Suitable genetically encoded voltage sensors might help examine such phenomena, and recent work towards the attainment of such sensors has evolved on multiple fronts based on fluorescent proteins and microbial opsins.

An early genetically encoded voltage sensor used the Shaker K^+^-channel as the voltage-sensitive domain (VSD), but subsequent work focused on the *Ciona* *intestinalis* VSD (Ci-VSD) [2,9]. For example, the combination of Ci-VSD and red-shifted fluorophores led to FRET voltage sensors (VSFP2.x, VSFP-mUKG-mKOκ, and VSFP-clover-mRuby variants) with ~20-100 ms kinetics and superior sensitivity compared to prior sensors [10–15]. Other sensors based on pH-sensitive GFP, circularly permuted GFP, and hybrid Ci-VSD domains yielded further enhancements in dynamic range and kinetics [16–20]. However, despite the fact that Ci-VSD based sensors have used bright fluorescent proteins, they have yet to realize the large dynamic range and fast kinetics of the small molecule voltage-sensitive dyes (e.g. [3–6]). Ci-VSD based sensors generally have a modest response (Δ*F*/*F* < 3%) to neural action potentials.

Another recent class of voltage sensors has employed rhodopsin family proteins, which contain both a voltage-sensitive domain and a fluorophore. Work on the bacteriorhodopsin photocycle has revealed that rhodopsin acts as a proton pump by absorbing a photon and using the energy for isomerization of the bound retinal. The subsequent conformational and electronic rearrangements induce transfer of a proton from the inside to the outside of the cell [21,22]. Archaerhodopsin-3 (Arch) is structurally homologous to bacteriorhodopsin and pumps a hyperpolarizing current when excited optically [23].

The initial state of Arch containing the isomerized retinal and deprotonated Schiff base is voltage-sensitive, and trans-membrane voltage modulates absorption in both proteorhodopsin and Arch [24,25]. This in turn confers a modulation to Arch’s weak fluorescence. Hence, fluorescence in wild-type Arch reports membrane voltage dynamics with sub-millisecond response times. However, the simultaneous photocurrent generated by the imaging illumination can alter neuronal function. A mutation of Arch (D95N) eliminated the photocurrent and demonstrated greater dynamic range (~40% per 100 mV) but also exhibited slow kinetics with a ~41 ms rise time at room temperature [25]. These Arch voltage sensors are also relatively dim, exhibiting two to three orders of magnitude lower quantum yield than GFP.

Improvements to voltage sensors performance hinge on increases in brightness as well as Δ*F/F* responses to voltage activity, which both contribute to optical detection capabilities [26]. The sensors without photocurrent from the Ci-VSD class and the Arch class all exhibit limited responses to action potentials (Δ*F*/*F* < 5%), as their slow rise times of tens of milliseconds prevent the sensors from taking advantage of their large (steady-state) dynamic range (~40% per 100 mV). Thus, faster kinetics, leading to increases in Δ*F/F* responses to fast voltage transients, could significantly improve the prospects of using such genetically encoded voltage sensors for imaging action potentials.

Here we present new Archaerhodopsin fluorescent voltage sensors with faster kinetics and larger dynamic range than Arch-D95N. We also show that improved trafficking signals improve the membrane localization of Arch sensors, which promotes superior optical readouts of membrane voltage. We then describe new mutations of Arch that improve its kinetics and dynamic range. By using signal detection theory to analyze our experimental measurements, we show that these improvements yield a sensor that reports neuron action potentials with up to ~3 times the spike detection fidelity of Arch-D95N — without photocurrent induced by the imaging illumination.

## Results

### *Enhanced trafficking motifs improved Arch membrane localization*

As a first step in enhancing voltage sensor performance, we sought to improve the sensor’s targeting to the cell membrane. In the original study of Arch’s capabilities as a voltage sensor, computational weighting of image pixels was necessary to separate useful fluorescence responses to changes in trans-membrane potential from non-responsive, background fluorescence [25]. Much of the latter fluorescence likely arose from mis-targeted Arch molecules. By using a high optical magnification and then heavily weighting pixels at the cell membrane, it was possible to largely disregard the majority, non-responsive pixels. However, if one wishes to image many cells over a wide field of view, this strategy of highly magnifying the image of each neuron is not viable.

To enhance the membrane localization of Arch sensors, we employed the same trafficking motifs used previously with a broad class of inhibitory opsins [27,28]. Prior work had shown that the endoplasmic reticulum (ER) export sequence (FCYENEV) and the Golgi export trafficking signal (TS) sequence (KSRITSEGEYIPLDQIDINV), found in membrane localized Kir2.1 potassium channels [29], improved NpHR membrane localization when added to the appropriate termini of the opsin protein. The resulting enhanced opsins had 3–5 times greater photocurrent than counterparts without the trafficking motifs, with fewer intracellular aggregates [27,28,30]. Although reports that another Kir 2.1 export motif improved the membrane localization of other voltage sensors such as VSFP-butterfly [20], this export motif did not notably improve rhodopsin membrane localization in previous studies [28].

**Figure 1:**
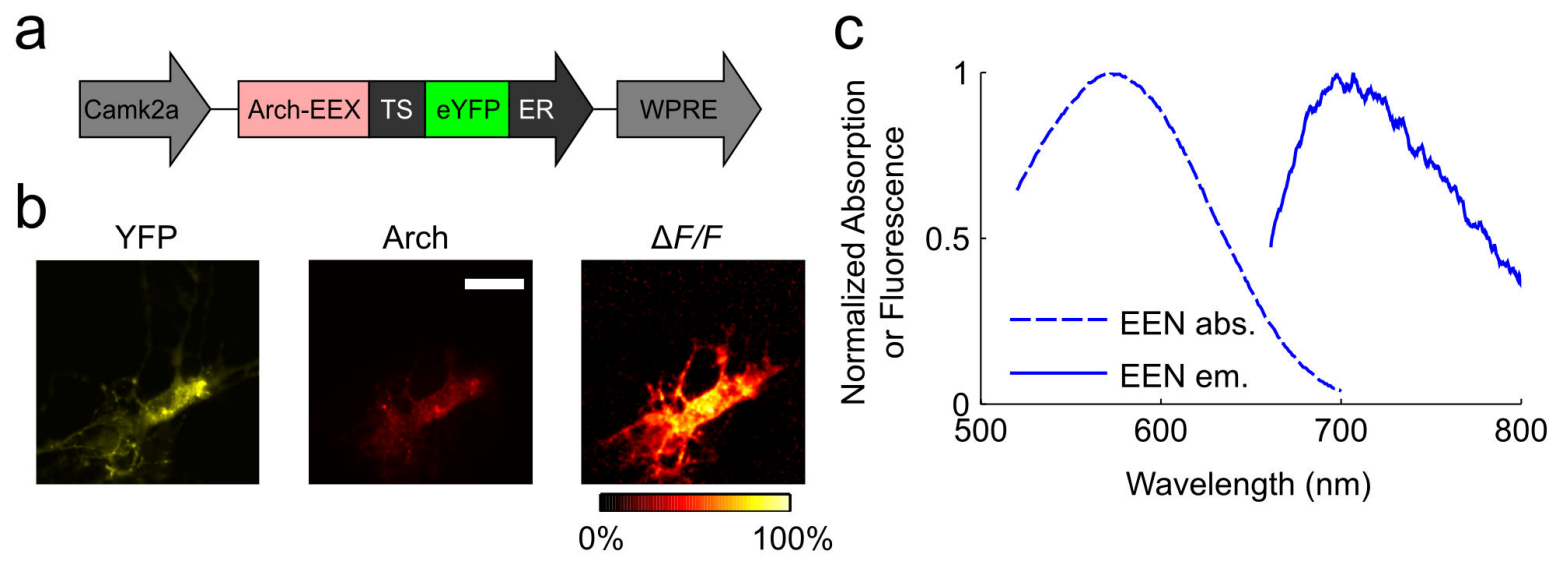
Fluorescence spectra and membrane localization of enhanced Arch constructs. (**a**) We expressed the enhanced Arch constructs (EEQ and EEN versions) as a fusion with eYFP under the control of the *Camk2a* promoter and targeted the fusion protein to the cell membrane using the localization sequences TS and ER. (**b**) Fluorescence signals from a labeled neuron, as seen in the YFP (*left*) and Arch-EEN (*middle*) fluorescence channels, along with the normalized spatial map (*right*) of the fluorescence response to a voltage step depolarization. Regions in which YFP fluorescence was visible also generally showed responses by Arch to voltage depolarization, which is indicative of successful membrane targeting. Scale bar is 20 µm. (**c**) Absorption and fluorescence spectra of Arch-EEN. The spectra of Arch-EEQ are similar.

We generated all of the Arch variants discussed in this paper within a similar construct containing the CaMKIIα (*Camk2a*) promoter and the woodchuck hepatitis post-transcriptional regulatory element (WPRE) (Figure 1a). In neurons transfected with constructs containing the trafficking signals, YFP, and Arch fluorescence signals were generally membrane-localized, indicative of minimal intracellular aggregation (Figure 1b).

We applied a voltage step protocol to neurons expressing Arch variants while we monitored fluorescence emissions (Methods). To qualitatively assess the degree of membrane localization, we computed the changes in fluorescence intensity Δ*F/F* across entire images (Figure 1b). The pixels with the greatest Δ*F/F* values were located at the outer edges of the cell image, but we also observed substantial Δ*F/F* values for pixels within the cell’s interior. These pixels included fluorescence signals from parts of the cell membrane that were outside the focal plane, but from which we nonetheless captured fluorescence emissions due to the lack of optical sectioning. As expected, interior pixels exhibited lower emission intensities but only moderately lower Δ*F/F* values than pixels on cells’ outer edges. These observations suggest the trafficking signals improved membrane localization and reduced voltage-insensitive Arch protein aggregation in the cell body. Given this improved localization, we binned pixels to much lower resolution than in previous studies, ranked pixels based on their signal-to-noise ratio (SNR), and selected a sufficient number of pixels to cover the cell soma (Methods). Such coarse-grained analyses are compatible with imaging studies over a large field-of-view, while preserving the dynamic range of the experimentally measured Δ*F/F* values.

### *Arch mutants exhibited faster kinetics and larger dynamic range than Arch-D95N*

To enhance Arch’s voltage sensitivity, we drew on the extensive literature regarding how protein mutations affect the bacteriorhodopsin photocycle. Using the homology between bacteriorhodopsin and Arch in their amino acid sequences, we noted that the proton pumping process translocates a proton through the D95, D222, E214, and D106 positions of Arch [31–33]. The deprotonation of the Schiff base and donation of charge to the D95 position is the first proton translocation step of the photocycle. Reprotonation of the Schiff base, with charge donated from the D106 position, is the final step [21,22]. Because these two steps are key to modulating the charge state of the Schiff base, and thereby the absorption and fluorescence, we considered mutations at these positions to enhance Arch’s voltage sensitivity.

Mutating the D95 amino acid to an uncharged amino acid is a general way to eliminate photocurrent [25,31–33]. Besides the aforementioned D95N mutation, D95S and D95T mutations at the homologous position in bacteriorhodopsin led to a Cl^-^ current, which would not benefit voltage imaging [34]. In recent reports, the mutation in bacteriorhodopsin homologous to D95Q also eliminated photocurrent [35]. We examined this mutation in Arch. When expressed in HEK cells, this mutation yielded 50% Δ*F/F* per 100 mV change in membrane voltage and no photocurrent, but it also exhibited slow kinetics, with a ~70 ms on-time, much like Arch-D95N.

Mutations to positions homologous to the Arch-D106 in bacteriorhodospin also modified the photocycle. Because D106 serves as the donor to the Schiff base during the reprotonation step, changing D106 to an uncharged amino acid slowed the photocycle and reduced the photocurrent [33]. We initially employed the mutation D106N in Arch in hopes of reducing photocurrent, but the mutant demonstrated non-negligible photocurrent without voltage sensitivity (data not shown). This observation suggested that D106 is the primary conduit for electrons to protonate and deprotonate the voltage-sensitive Schiff base during modulations of membrane voltage. Thus, mutation of the aspartic acid to the homologous glutamic acid could shift the relative pK_a_ between the D106 position and the Schiff base, altering the kinetics of protonation and deprotonation. This combination of observations on the D95 and D106 positions led us to try the double mutations D95N-D106E (Arch-EEN) and D95Q-D106E (Arch-EEQ).

**Figure 2:**
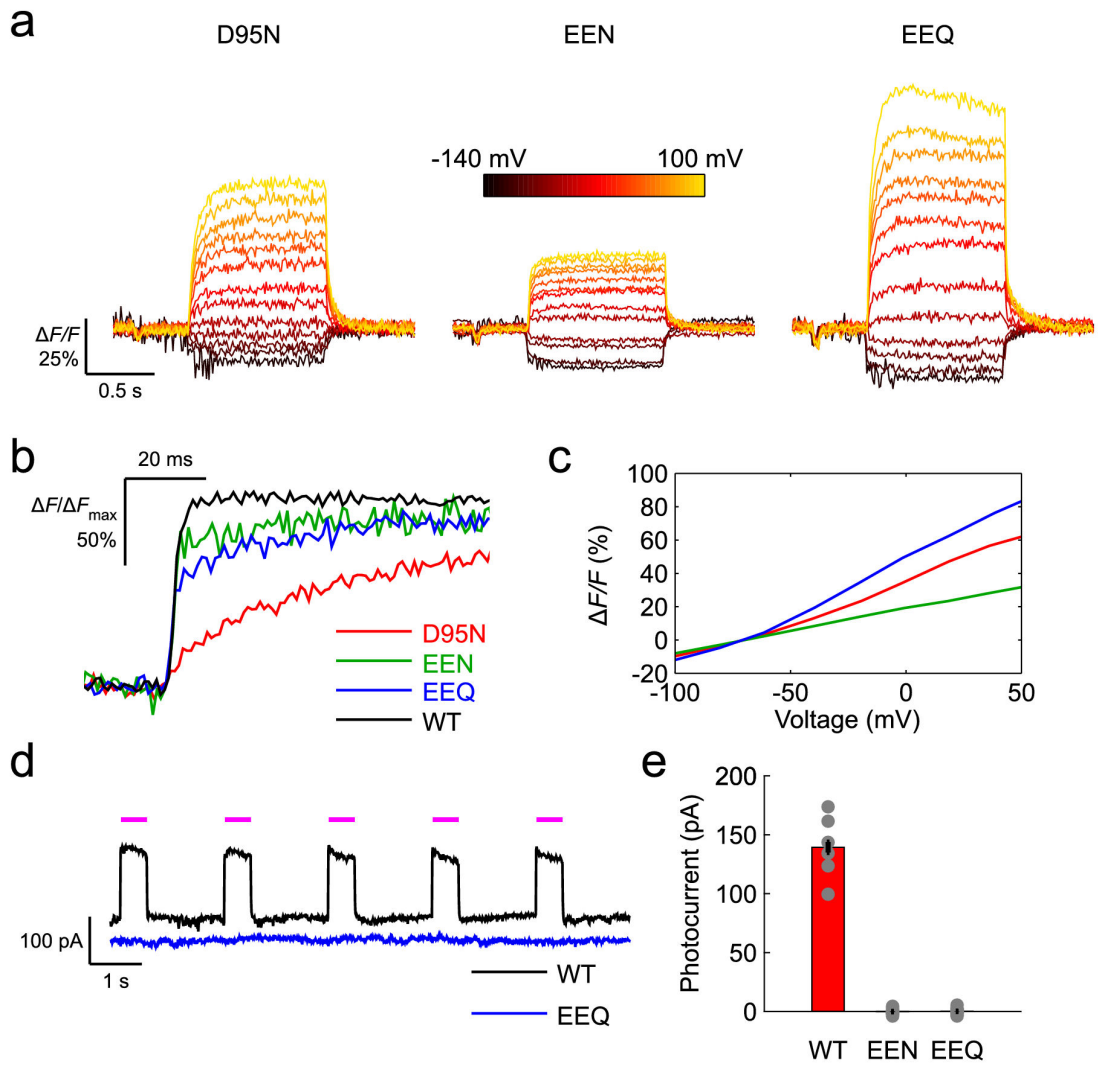
Enhanced Arch variants respond more quickly and with greater dynamic range to voltage depolarization. (**a**) Optical step responses of neurons transfected with Arch-D95N, Arch-EEN, and Arch-EEQ, imaged at 440 Hz. We held neurons at ‒ 70 mV at the start of each trace and stepped to command voltages ranging from ‒ 140 mV to +100 mV. (**b**) Optical step response of Arch constructs expressed in neurons when raising the voltage from ‒ 70 mV to +30 mV, normalized to each sensor’s maximum step response and displayed over shorter time scales than in (**a**), imaged at 1000 Hz. (**c**) Steady state fluorescence response of Arch constructs as a function of membrane voltage. (**d**) Arch-WT generated appreciable photocurrent with 633 nm illumination (magenta bars), whereas Arch-EEQ generated negligible photocurrent. (**e**) Steady-state photocurrents of various Arch constructs in response to the same 633 nm illumination. Error bars represent standard error to the mean (s.e.m). Illumination intensity at the specimen was 1400 mW/mm^2^ for all panels.

Spectrally, the two Arch mutants (which we will term Arch-EEx, when referring to them both) are similar to Arch-D95N in both absorption and emission (Figure 1c). Arch-EEx had greater dynamic range than Arch-D95N in response to various voltage and current waveforms (Methods). First, we compared the step response of Arch-D95N, Arch-EEN, and Arch-EEQ in neurons at low frame rate 440 Hz (Figure 2a). We then examined the initial rise of each mutant’s optical waveform by imaging with high frame rate (1000 Hz) the fluorescence response to a voltage depolarization from ‒ 70 mV to +30 mV (Figure 2b). The D106E mutation yielded fast kinetics for both the D95N and D95Q mutations, resulting in a ~5–15 ms rise time to within 1/*e* of the full response, as compared to ~45 ms for Arch-D95N (Methods). We also observed a fast component of the fluorescence rise for both Arch-EEN and Arch-EEQ. This fast component improved the Δ*F/F* in response to action potentials, as described below. We averaged the steady state Δ*F/F* as a function of the applied voltage (Figure 2c), and observed that Arch-EEQ outperformed Arch-D95N, reaching 60% Δ*F/F* per 100 mV. Arch-EEN was inferior to Arch-D95N in this regard, yielding only 20% Δ*F/F* per 100 mV voltage step.

Because wild-type Arch generates inhibitory photocurrent that modifies neuron function, we also checked whether the new mutants produced any photocurrent. We observed substantial photocurrent for a neuron transfected with wild-type Arch during the application of the excitation laser at the same illumination intensities (1400 mW/mm^2^) used during imaging (Figure 2d). However, under identical optical conditions we recorded little photocurrent for a neuron transfected with Arch-EEQ. By averaging the measured photocurrent over many cells, we observed negligible photocurrent for both Arch-EEN (-0.1 pA ± 0.1 pA, *n* = 10 cells) and Arch-EEQ (0.2 pA ± 0.1 pA, *n* = 16 cells) (Figure 2e).

**Figure 3:**
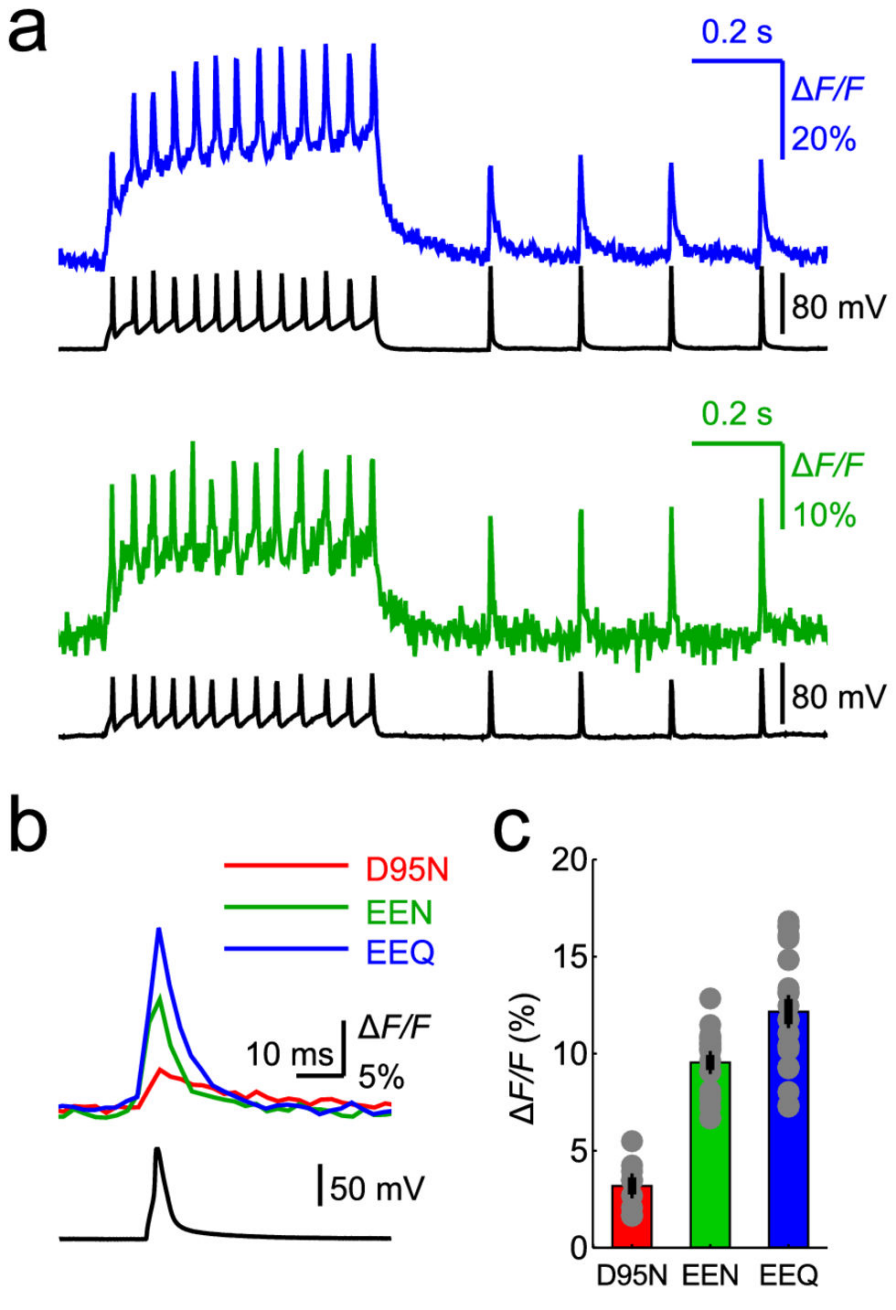
Enhanced Arch sensors report single action potentials with greater peak amplitude than Arch D95N. (**a**) Fluorescence traces from a neuron expressing Arch-EEQ (*top*) and a neuron expressing Arch-EEN (*bottom*) showed sharp peaks corresponding to action potentials in the simultaneous electrophysiology measurements. (**b**) The average optical waveforms (average of *n* = 20, 20, and 12 traces for the EEQ, EEN, and D95N traces, respectively) of the Arch sensors’ response (*top*) were similarly shaped to the average electrical waveform of individual action potentials (*bottom, n* = 20 traces). (**c**) Average peak responses to single action potentials for Arch-D95N (*n* = 7 cells), Arch-EEN (*n* = 20 cells), and Arch-EEQ (*n* = 23 cells). Error bars are s.e.m. The fluorescence imaging rate was 440 Hz, and the illumination intensity was 1400 mW/mm^2^ for all panels.

The faster kinetics of the Arch-EEx mutants also improved the fluorescence responses to neuronal action potentials. Typical optical traces (frame rate 440 Hz) corresponded well to simultaneously recorded traces of electrical activity, regarding both spiking and sub-threshold dynamics (Figure 3a). Notably, the Arch-EEx sensors’ fast and slow response components served distinct functions. The fast component facilitated spike detection, whereas the combination of the fast and slow components relayed the fluctuations in baseline voltage. We computed the Δ*F/F* of individual neurons in response to single action potentials induced by short current injections (Figure 3b-c). The electrophysiological recordings for cells expressing Arch-EEQ, -EEN, and -D95N respectively revealed FWHM action potential widths of 3.5 ± 0.8 ms, 3.4 ± 0.6 ms, and 3.1 ± 0.8 ms (mean ± standard deviation; *n* = 7–23 cells) (Figure 3b), which are typical values for cultured neurons. Due to its faster kinetics, Arch-EEN exhibited a ~3-fold increase in peak Δ*F/F* in response to neural spikes as compared to Arch-D95N, despite Arch-EEN’s reduced steady-state dynamic range. Similarly, Arch-EEQ had a 4-fold increase in peak Δ*F/F* response to spikes versus Arch-D95N, with Δ*F/F* values as high as 17% for single cells. It is likely that improved pixel selection and background subtraction could yield even higher Δ*F/F* values.

### *Arch mutations improved spike detection fidelity*

**Figure 4:**
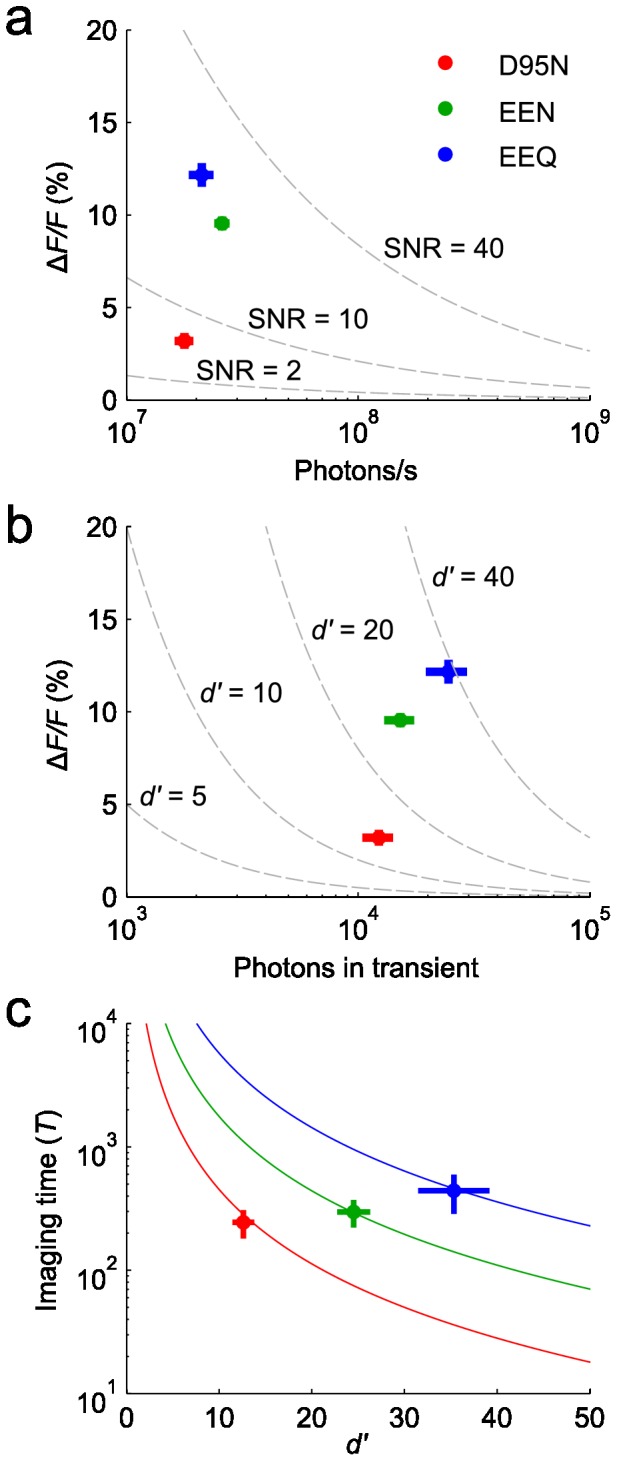
Enhanced Arch sensors outperformed Arch-D95N in several measures characterizing detection of neuronal spikes. (**a**) Peak Δ*F/F* values of the optical responses to action potentials plotted as a function of the average photon emission rate (F_0_) during the baseline periods, as determined from the experimental optical waveforms. The dashed lines represent iso-contours of SNR at the time of the emission peak, defined as the ratio of the peak response to the standard deviations in the baseline shot noise, $(\Delta F/F)\times \sqrt{F_{0}/\nu }$. Arch-EEQ outperformed Arch-D95N by a factor of ~4, while Arch-EEN outperformed Arch-D95N by a factor of ~3. (**b**) Peak Δ*F/F* values of the optical responses to action potentials plotted as a function of the estimated total number of photons detected per spike. Dashed lines represent iso-contours of spike detection fidelity, *d*’, as defined in Wilt et al. 2012 and as determined from the experimentally measured optical waveforms. Arch-EEQ outperformed Arch-D95N by a factor of ~2.8. (**c**) The enhanced Arch sensors enabled longer experimental imaging durations above minimum values of detection fidelity. For experiments that start with an initial value of *d*’, the solid lines plot the total imaging time (*T*) that the sensors can provide a spike detection fidelity >*d*’*/e*. For initial value of *d’* shown, Arch-EEQ achieved total imaging durations an order of magnitude longer than those achieved by Arch-D95N. We obtained all imaging data at a frame rate of ν = 440 Hz and an illumination intensity of 1400 mW/mm^2^. All data points represent mean ± s.e.m.

We characterized the new Arch mutants using various measures of SNR and spike detection capabilities, and found that the sensors outperformed Arch-D95N. A simplistic notion of SNR is the peak response divided by the standard deviation of the baseline fluorescence in one time bin (Methods). We compared the shot-noise limited performance of the various sensors using this metric, all at the same imaging conditions (440 Hz frame rate; 1400 mW/mm^2^ illumination) (Figure 4a). Because of the similar brightness of all three mutants under 633 nm excitation, the new Arch mutants had similar increases in SNR, as predicted from the increases in peak Δ*F/F* responses.

We also used a signal detection theoretic framework to quantify the fidelity of spike detection [26]. The spike discriminability described in Ref. 26, denoted here as *d*’, indexes the receiver-operating characteristic (ROC) curve for spike detection and quantifies the degree to which spikes can be correctly identified within fluorescence data attained at the physical noise limits set by photon shot noise (Methods). This figure of merit takes into consideration a neuron’s experimentally determined fluorescence baseline, the peak Δ*F/F* response to a spike, and the temporal duration of the sensor’s response to a spike. By aggregating photons over multiple frames and accounting for sensor kinetics, this framework provides a far more realistic measure of spike detection fidelity than more traditional, simplistic measures of SNR such as that shown in Figure **4a**.

To compare spike discriminability, we combined onto one plot the iso-contours of *d*’, the sensors’ peak Δ*F/F* values, and the total number of photons aggregated within a fluorescent transient (Figure 4b). Despite the inferior peak Δ*F/F* value of Arch-D95N, the slow kinetics of Arch-D95N provided partial compensation when comparing its *d’* value to those of Arch-EEx; Arch-D95N’s slower kinetics yielded longer transients that increased the total photon count associated with action potentials. However, Arch-EEQ and Arch-EEN still outperformed Arch-D95N, by factors of 2.8 and 2.0 in *d*’, respectively.

Another way to assess voltage sensors’ efficacy is to compare the total durations over which they allow reliable spike detection before undergoing photobleaching. We determined this duration, *T*, for the various Arch sensors by examining the photobleaching kinetics (Figure 4c) (Methods). Increasing illumination intensity improves *d’* by increasing the photon emission rate, but it also reduces *T* by speeding photobleaching. We characterized this trade-off between *d’* and *T* across varying illumination intensities by defining a constant ‘photon capacity’ for each sensor as the product of the sensor’s photobleaching time and initial photon emission rate (Figure 4c). Because *d’* is proportional to peak Δ*F/F*, any improvement in the latter can yield corresponding percentage increases in *T* if one reduces the illumination intensity to achieve a fixed *d’* value. The *T* value for Arch-EEQ was greater than that of Arch-D95N by an order of magnitude, at any fixed *d’* (Figure 4c).

### *Arch is compatible with multi-spectral excitation and imaging*

The red-shifted absorption and fluorescence spectrum of Arch were compatible with simultaneous excitation of other proteins with cyan or green light. We employed the 2A linker, which allows the two parts of a bicistronic sequence to be expressed as separate protein molecules by breaking protein translation at the 2A linker position [36,37]. This approach enabled us to express Arch-EEQ along with another protein of interest in the same set of cells. Polypeptide linkers and IRES sequences also enable expression of two proteins using only one construct in either fusion or separated forms. However, the 2A linker permits stoichiometric expression but distinct patterns of localization for the two protein species, for each protein retains its own trafficking motifs [36,37]. As illustrations, using the p2A self-cleaving linker we made constructs that simultaneously express Arch with ChR2 [38] and GCaMP5 [39]. Prior work has shown that an NpHR-2A-ChR2 construct yielded both significant excitatory and inhibitory current, depending on the excitation wavelength, and that multiple membrane proteins can express in one cell [28].

**Figure 5:**
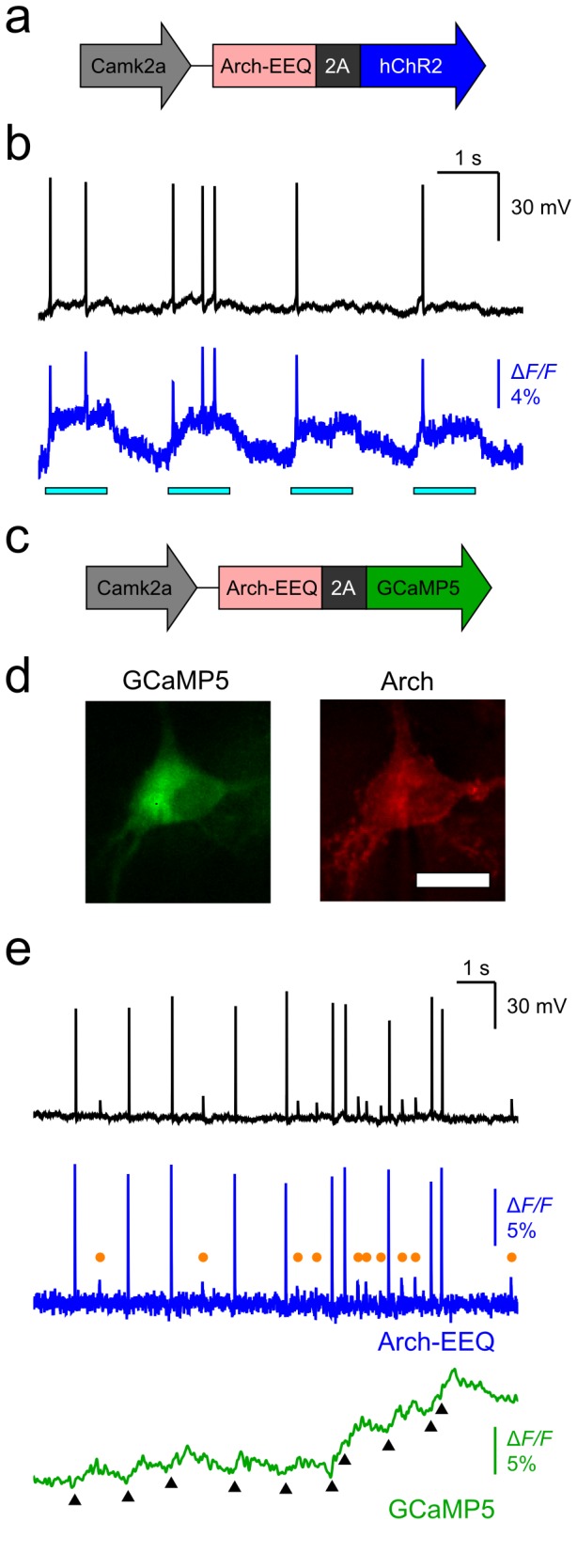
Bicistronic constructs expressing Arch-EEQ with either ChR2 or GCaMP5 enabled voltage imaging in combination with either optogenetic control or calcium imaging. (**a**) Schematic of the Arch-2A-hChR2 construct. (**b**) The Arch-2A-hChR2 construct enabled all-optical stimulation and readout of neurons. We imaged neurons transfected with the Arch-2A-hChR2 construct and simultaneously measured the transmembrane potential (black trace) and Arch fluorescence (blue trace). Photoexcitation of ChR2 (horizontal cyan bars; λ = 488 nm, 4 mW/mm^2^) elicited spikes in both the optical and electrical measurements. (**c**) Schematic of the Arch-2A-GCaMP5 construct. (**d**) We observed neurons transfected with the Arch-2A-GCaMP5 construct by simultaneously imaging GCaMP5 fluorescence (green channel, cytosol localized) and Arch fluorescence (red channel, membrane localized). Scale bar is 20 µm. (**e**) The Arch-2A-GCaMP5 construct enabled simultaneous measurements of voltage (blue trace) and intracellular calcium (green trace). Although action potentials were apparent in both the Arch and GCaMP5 signals (black triangular markers in the GCaMP5 trace; λ = 488 nm excitation, *I* = 10 mW/mm^2^), Arch signals were far superior in reporting brief, sub-threshold depolarizations (orange circles in the Arch trace). The fluorescence imaging rate was 440 Hz, and the illumination intensity for Arch excitation (λ = 633 nm excitation) was 1400 mW/mm^2^ for all panels.

First, we made an Arch-EEQ-2A-hChR2 construct, to enable simultaneous optical control and readout of neuronal voltage (Figure 5a). Under similar illumination as above, we simultaneously imaged the Arch sensor (633 nm excitation; 1400 mW/mm^2^) and depolarized the neuron (cyan bars; 488 nm excitation; 4 mW/mm^2^) (Figure 5b). Electrical recordings confirmed that action potentials occurred during the cyan stimulation. Second, we made an Arch-EEQ-2A-GCaMP5 construct, for simultaneous readouts of a neuron’s voltage and calcium dynamics (Figure 5c). Arch was membrane localized, whereas GCaMP5 expression was cytosolic, as expected with this construct (Figure 5d). This segregated localization pattern might facilitate separation of signals from the two fluorescence channels. We triggered action potentials by injecting brief current pulses and observed the ensuing voltage and calcium dynamics concurrently (Figure 5e). Since some of the current pulses failed to initiate action potentials, these induced only sub-threshold voltage depolarizations in the membrane potential (Methods). The Arch fluorescence trace reported these sub-threshold events (orange markers), but the GCaMP5 fluorescence trace did not.

## Discussion

We have presented an Arch voltage sensor that outperforms the previously published version by a factor of 2.8 in *d*’. Direct comparisons to other classes of genetically encoded voltage sensors (mostly based on variants of GFP) are difficult, as each class of sensor has different excitation and emission wavelengths, and are tested under different excitation power levels. These three factors contribute to the level of background fluorescence, optical scattering, and phototoxicity, which are all important parameters to consider regarding the utility of voltage sensors in brain slice and live animal preparations.

Although voltage sensors based on Ci-VSD have exhibited large steady state Δ*F/F* values (40% per 100 mV), slow rise times have limited optical responses to spikes to ~3% fluorescence changes. In brain slices and *in vivo* preparations, background and autofluorescence emissions are far more substantial than in cultured neurons, causing further degradation of usable signals. Excitation powers in the tens of mW/mm^2^ are needed to detect spikes with *d’* >10, forcing severe tradeoffs between having sufficient signal but avoiding photobleaching. Since voltage sensors based on Ci-VSD generally already have fluorophores of high quantum yield (>10%) attached to the VSD, it is likely that improvements in the effective peak Δ*F/F* will be needed for this class of sensor to improve voltage imaging of single spikes in brain slice or *in vivo*, either by improving SNR or reducing the requisite illumination levels.

By comparison, Arch suffers from low quantum yield but has a substantial peak Δ*F/F* response. The excitation power densities used here with Arch enable single cell studies in culture, but do not easily permit wide-field fluorescence imaging in brain slices, for which the high illumination intensity is likely to yield substantial background auto-fluorescence. In fact, studies employing *in utero* electroporation with Arch constructs have already reported that the fluorescence signals were negligible in comparison to background auto-fluorescence [2]. Thus, researchers should improve upon the low quantum yield of Arch, either by direct mutation to the Arch chromophore or by indirect approaches such as using FRET excitation. Regardless of Arch’s emission properties, we have shown that Arch detects voltage transients with relatively large dynamic range and fast kinetics compared to existing fluorescent protein voltage sensors, and thus serves as a viable alternative to Ci-VSD as a voltage sensitive domain.

Voltage sensors are likely to play different roles than calcium sensors in neuroscience research. The most recent genetically encoded calcium sensors are approaching reliable, single spike sensitivity, although their slow kinetics limits them to detecting temporally sparse action potentials [39]. Still, this form of spike detection could be sufficient for studying the sparse spiking dynamics of important cell classes, such as neocortical pyramidal neurons. Genetically encoded voltage sensors may find niche applications in the study of fast-spiking, sub-threshold, or population-level dynamics in genetically defined neuron types, all of which calcium sensors poorly report [40]. Many neuronal networks contain fast-spiking inhibitory neurons that regulate other cells’ sub-threshold voltages, leading to rich classes of oscillatory activity. As revealed by our simultaneous voltage and calcium imaging studies, voltage sensors with sufficiently fast kinetics (rise time <10 ms) and large Δ*F/F* (50% per 100 mV) can detect sub-threshold activity that is missed by calcium sensors. Thus, imaging studies of fast spiking and sub-threshold dynamics using with the new generation of voltage sensors might contribute to the understanding of networks’ excitatory-inhibitory balance and oscillatory phenomena.

## Methods

### *Ethics Statement*

The Stanford University Administrative Panel on Laboratory Animal Care (APLAC) approved all animal experiments.

### *Arch constructs*

Arch mutants originated from Arch-GFP (Addgene plasmid 22217). We constructed Arch-EEN, Arch-EEQ, and enhanced Arch-D95N by site-directed mutagenesis in Arch, and subsequent overlap assembly with eYFP and trafficking signals. We removed the eYFP portion of the Arch-EEQ construct when making the Arch-2A constructs. The Arch variants are deposited on Addgene as plasmids 45188 (Arch-EEQ) and 45189 (Arch-EEN).

### *Primary cell culture*

We dissected rat hippocampal cells from Sprague-Dawley pups (postnatal day 0, Charles-River Labs) and cultured them in Neurobasal media (Invitrogen) supplemented with glutamine and B27 (Invitrogen) [41]. We transfected Arch mutant plasmid DNA using calcium phosphate 3–5 days after plating and imaged the neurons 3–5 days after transfection.

### *Electrophysiology*

We simultaneously obtained fluorescence measurements and voltage or current clamp measurements at 22°C while holding neurons in a perfusion chamber mounted on the microscope stage. The extracellular solution contained 150 mM NaCl, 4 mM KCl, 10 mM glucose, 10 mM HEPES, 2 mM CaCl_2_, and 2 mM MgCl_2_. The intracellular solution contained 129 mM K-gluconate, 10 mM KCl, 10 mM HEPES, and 4 mM Na _2_ATP. We pulled patch pipettes made of borosilicate glass pulled to resistances of 3-6 MΩ (Sutter Instruments P-97), yielding access resistances of 5-25 MΩ in whole-cell mode.

We patched neurons using an Axopatch 700B amplifier (Axon Instruments), and used pClamp software (Axon Instruments) to generate the various control waveforms and to record voltage and current traces. The voltage control waveform applied a holding potential of -70 mV at the start of each cycle and then applied steps lasting 1 s to voltages ranging from -140 mV to +100 mV in 20 mV increments. To generate action potentials in current clamp, we injected current using both long pulses (10–200 pA; > 0.5s) or short pulses (300-1000 pA; 2 ms). The short current pulses triggered spikes with near unity efficiency when tuned above the spiking threshold, and triggered spikes with ~50% efficiency when near the spiking threshold. We corrected *post hoc* the membrane voltage for the junction potential and access resistance.

### *Fluorescence imaging*

We used an epi-fluorescence microscope (Olympus IX51) with a 40× 0.8 NA water immersion objective for time-lapse imaging. We used a 480 nm LED (89 North Heliophore) or a 633 nm HeNe laser; these sources passed through an excitation filter, either 475/25 nm or 620/40 nm, for GFP/GCaMP/YFP and Arch excitation, respectively. We imaged all Arch fluorescence traces using an illumination of 1400 mW/mm^2^ at the specimen plane. Fluorescence emissions passed through a 495 nm dichroic mirror and a long-pass emission filter for GFP/GCaMP/YFP imaging, but through a 645 nm dichroic mirror and a 697/75 nm emission filter for Arch. Dual color experiments used a quad-band 405/488/561/635 nm excitation filter (Semrock) and a DualView emission splitter (Optical Insights). The latter split the green emission and arch emission using a 565 nm dichroic mirror; the two resulting fluorescence channels had 535/50 nm and 697/75 nm emission filters, respectively. An Andor iXon 897 electron-multiplied charge-coupled device camera cooled to -80°C recorded the emission at 400–1000Hz, using binned pixels of 3 µm × 3 µm (or larger) in the image plane to achieve the image-acquisition speed.

### *Determination of ΔF/F maps*

We computed Δ*F/F* maps as the difference between the mean fluorescence with and without the applied voltage step, for each pixel. We ranked pixels according to their SNR, defined here as $\left(\Delta F/F\right)\times \sqrt{\bar{F}}$, where Δ*F/F* is the change in normalized fluorescence when applying the voltage step, and $\bar{F}$ is the mean fluorescence of the pixel for the entire trace. We then aggregated the fluorescence from the top 20% image pixels to produce fluorescence time traces for further analysis. This subset of pixels was sufficient to cover the cell soma.

### *Absorption and emission spectra*

We obtained absorption and emission spectra by transferring Arch mutants to plasmids with the CMV promoter and transfecting HEK cells at a total volume of 100 mL for 3 days. We added all-trans retinal (5 µM) and cultured the cells in the dark for 3 h. We pelleted the cells, lysed them using a sonicator in 50 mM Tris with 2 mM MgCl_2_ at pH 7.3, and pelleted again. Finally, we homogenized the lysate in PBS (pH 7.2) supplemented with 1.5% dodecyl maltoside and pelleted once more. We obtained the absorption spectrum using the supernatant and a spectrophotometer (Tecan Safire 2 UV–Vis). We obtained the emission spectrum using the same imaging setup for patch experiments, a spectrometer (Thorlabs CCS200), and 633 nm excitation.

### *Determinations of photobleaching rate and photon capacity*

We re-used the same traces in the patch-clamp experiments to determine photon counts and bleaching decay times, from which we computed the total number of photons emitted by a cell before photobleaching. By performing exponential fits to resting periods in the fluorescence traces without electrophysiological manipulations, we determined the photobleaching time-constant for each trace. We computed the photon capacity as the product of the initial photon emission rate and the photobleaching time (9).

### *Determination of spike detection fidelity*

Because we imaged all Arch samples with the same illumination intensity (*I* = 1400 mW/mm^2^), we were able to compare directly the photon emission rates of the different sensors. We first defined SNR for spike detection in a shot-noise limited optical trace as SNR=$\left(\Delta F/F\right)\times \sqrt{F_{0}/\nu }$, where *F*_0_ is the photon emission rate for a neuron and ν is the data sampling rate. This SNR is the peak response Δ*F* divided by the standard deviation of the baseline ($\sqrt{F_{0}/\nu }$). We also computed iso-contours of SNR using this relation.

In addition, we used a signal detection framework to compute the spike discriminability parameter, *d*’ (20). This characterizes a sensor’s spike detection fidelity in a way that considers both the duration and the intensity of the fluorescence signal in response to action potentials. We used experimental determinations of the mean fluorescence intensity and the average fluorescence waveform in response to single action potentials (e.g. Figure 3b) to compute *d*’. We computed iso-contours of *d’* by approximating the fluorescence transients as mono-exponential decays, and computing the initial value of *d’* from Δ*F/F* and the estimated total number of photons within the transient (20). For experiments with an initial value *d*’, we defined the imaging time (*T*) as total experimental duration that the sensor provides a spike detection fidelity >*d*’*/e*.

## References

[1] Peterka DS, Takahashi H, Yuste R (2011) Imaging voltage in neurons. Neuron 69: 9–21. doi:10.1016/j.neuron.2010.12.010. PubMed: 21220095. 21220095 10.1016/j.neuron.2010.12.010 PMC3387979

[2] Mutoh H, Akemann W, Knöpfel T (2012) Genetically engineered fluorescent voltage reporters. Acs Chem Neurosci 3: 585-592. doi:10.1021/cn300041b. PubMed: 22896802. 22896802 10.1021/cn300041b PMC3419450

[3] Grinvald A, Hildesheim R (2004) VSDI: a new era in functional imaging of cortical dynamics. Nat Rev Neuro 5: 874-885. doi:10.1038/nrn1536. PubMed: 15496865. 10.1038/nrn1536 15496865

[4] Chemla S, Chavane F (2010) Voltage-sensitive dye imaging: Technique review and models. J Physiol Paris 104: 40-50. doi:10.1016/j.jphysparis.2009.11.009. PubMed: 19909809. 19909809 10.1016/j.jphysparis.2009.11.009

[5] Salzberg BM, Grinvald A, Cohen LB, Davila HV, Ross WN (1977) Optical Recording of Neuronal Activity in an Invertebrate Central Nervous System: Simultaneous Monitoring of Several Neurons. J Neurophysiol 40: 1281-1291. PubMed: 925730. 925730 10.1152/jn.1977.40.6.1281

[6] Salzberg BM, Obaid AL, Bezanilla F (1993) Microsecond Response of a Voltage-Sensitive Merocyanine Dye: Fast Voltage-Clamp Measurements on Squid Giant Axon. Japn J Physiol 43: Suppl. 1: S37-S41. 8271515

[7] Chanda B, Blunck R, Faria LC, Schweizer FE, Mody I et al. (2005) A hybrid approach to measuring electrical activity in genetically specified neurons. Nat Neurosci 8: 1619–1626. doi:10.1038/nn1558. PubMed: 16205716. 16205716 10.1038/nn1558

[8] Sjulson L, Miesenböck G (2008) Rational Optimization and Imaging In Vivo of a Genetically Encoded Optical Voltage Reporter. J Neurosci 28: 5582–5593. doi:10.1523/JNEUROSCI.0055-08.2008. PubMed: 18495892. 18495892 10.1523/JNEUROSCI.0055-08.2008 PMC2714581

[9] Siegel MS, Isacoff EY (1997) A genetically encoded optical probe of membrane voltage. Neuron 19: 735–741. doi:10.1016/S0896-6273(00)80955-1. PubMed: 9354320. 9354320 10.1016/s0896-6273(00)80955-1

[10] Sakai R, Repunte-Canonigo V, Raj CD, Knöpfel T (2001) Design and characterization of a DNA-encoded, voltage-sensitive fluorescent protein. Eur J Neurosci 13: 2314–2318. doi:10.1046/j.0953-816x.2001.01617.x. PubMed: 11454036. 11454036 10.1046/j.0953-816x.2001.01617.x

[11] Lundby A, Mutoh H, Dimitrov D, Akemann W, Knöpfel T (2008) Engineering of a genetically encodable fluorescent voltage sensor exploiting fast Ci-VSP voltage-sensing movements. PLOS ONE 3: e2514. doi:10.1371/journal.pone.0002514. PubMed: 18575613. 18575613 10.1371/journal.pone.0002514 PMC2429971

[12] Lundby A, Akemann W, Knöpfel T (2010) Biophysical characterization of the fluorescent protein voltage probe VSFP2.3 based on the voltage-sensing domain of Ci-VSP. Eur Biophys J 39: 1625–1635. doi:10.1007/s00249-010-0620-0. PubMed: 20686764. 20686764 10.1007/s00249-010-0620-0

[13] Mutoh H, Perron A, Dimitrov D, Iwamoto Y, Akemann W et al. (2009) Spectrally-resolved response properties of the three most advanced FRET based fluorescent protein voltage probes. PLOS ONE 4: e4555. doi:10.1371/journal.pone.0004555. PubMed: 19234605. 19234605 10.1371/journal.pone.0004555 PMC2641041

[14] Tsutsui H, Karasawa S, Okamura Y, Miyawaki A (2008) Improving membrane voltage measurements using FRET with new fluorescent proteins. Nat Methods 5: 683–685. doi:10.1038/nmeth.1235. PubMed: 18622396. 18622396 10.1038/nmeth.1235

[15] Lam AJ, St-Pierre F, Gong Y, Marshall JD, Cranfill PJ et al. (2012) Improving FRET dynamic range with bright green and red fluorescent proteins. Nat Methods 9: 1005–1012. doi:10.1038/nmeth.2171. PubMed: 22961245. 22961245 10.1038/nmeth.2171 PMC3461113

[16] Jin L, Han Z, Platisa J, Wooltorton JRA, Cohen LB et al. (2012) Single Action Potentials and Subthreshold Electrical Events Imaged in Neurons with a Fluorescent Protein Voltage Probe. Neuron 75: 779–785. doi:10.1016/j.neuron.2012.06.040. PubMed: 22958819. 22958819 10.1016/j.neuron.2012.06.040 PMC3439164

[17] Barnett L, Platisa J, Popovic M, Pieribone VA, Hughes T (2012) A Fluorescent, Genetically-Encoded Voltage Probe Capable of Resolving Action Potentials. PLOS ONE 7: e43454. doi:10.1371/journal.pone.0043454. PubMed: 22970127. 22970127 10.1371/journal.pone.0043454 PMC3435330

[18] Perron A, Mutoh H, Launey T, Knöpfel T (2009) Red-shifted voltage-sensitive fluorescent proteins. Chem. Biol. 16: 1268–1277. 10.1016/j.chembiol.2009.11.014 PMC2818747 20064437

[19] Mishina Y, Mutoh H, Knöpfel T (2012) Transfer of Kv3.1 Voltage Sensor Features to the Isolated Ci-VSP Voltage-Sensing Domain. Biophys J 103: 669–676. doi:10.1016/j.bpj.2012.07.031. PubMed: 22947928. 22947928 10.1016/j.bpj.2012.07.031 PMC3443783

[20] Akemann W, Mutoh H, Perron A, Park YK, Iwamoto Y, Knöpfel T (2012) Imaging neural circuit dynamics with a voltage-sensitive fluorescent protein. J Neurophysiol 108: 2323-2337. doi:10.1152/jn.00452.2012. PubMed: 22815406. 22815406 10.1152/jn.00452.2012

[21] Lanyi JK (2004) Bacteriorhodopsin. Annu Rev Physiol 66: 665-688. doi:10.1146/annurev.physiol.66.032102.150049. PubMed: 14977418. 14977418 10.1146/annurev.physiol.66.032102.150049

[22] Lanyi JK (1992) Proton transfer and energy coupling in the bacteriorhodopsin photocycle. J Bioenerget Biomembr 24: 169-179. doi:10.1007/BF00762675. PubMed: 1326515. 10.1007/BF00762675 1326515

[23] Chow BY, Han X, Dobry AS, Qian X, Chuong AS et al. (2010) High-performance genetically targetable optical neural silencing by light-driven proton pumps. Nature 463: 98–102. doi:10.1038/nature08652. PubMed: 20054397. 20054397 10.1038/nature08652 PMC2939492

[24] Kralj JM, Hochbaum DR, Douglass AD, Cohen AE (2011) Electrical spiking in Escherichia coli probed with a fluorescent voltage indicating protein. Science 333: 345–348. doi:10.1126/science.1204763. PubMed: 21764748. 21764748 10.1126/science.1204763

[25] Kralj JM, Douglass AD, Hochbaum DR, Maclaurin D, Cohen AE (2012) Optical recording of action potentials in mammalian neurons using a microbial rhodopsin. Nat Methods 9: 90–95. doi:10.1038/nchembio.1135. 10.1038/nmeth.1782 PMC3248630 22120467

[26] Wilt BA, Fitzgerald JE, Schnitzer MJ (2013) Photon shot-noise limits on optical detection of neuronal spikes and estimation of spike timing. Biophys J 104: 51-62. doi:10.1016/j.bpj.2012.07.058. PubMed: 23332058. 23332058 10.1016/j.bpj.2012.07.058 PMC3540268

[27] Gradinaru V, Thompson KR, Deisseroth K (2008) eNpHR: a Natronomonas halorhodopsin enhanced for optogenetic applications. Brain Cell Biol 36: 129–139. doi:10.1007/s11068-008-9027-6. PubMed: 18677566. 18677566 10.1007/s11068-008-9027-6 PMC2588488

[28] Gradinaru V, Zhang F, Ramakrishnan C, Mattis J, Prakash R et al. (2010) Molecular and Cellular Approaches for Diversifying and Extending Optogenetics. Cell 141: 154–165. doi:10.1016/j.cell.2010.02.037. PubMed: 20303157. 20303157 10.1016/j.cell.2010.02.037 PMC4160532

[29] Hofherr A, Fakler B, Klöcker N (2005) Selective Golgi export of Kir2.1 controls the stoichiometry of functional Kir2.x channel heteromers. J Cell Sci 118: 1935-1943. doi:10.1242/jcs.02322. PubMed: 15827083. 15827083 10.1242/jcs.02322

[30] Mattis J, Tye KM, Ferenczi EA, Ramakrishnan C, O’Shea DJ et al. (2011) Principles for applying optogenetic tools derived from direct comparative analysis of microbial opsins. Nat Methods 9: 159-172. doi:10.1038/nmeth.1808. PubMed: 22179551. 22179551 10.1038/nmeth.1808 PMC4165888

[31] Kolodner P, Lukashev EP, Ching YC, Rousseau DL (1996) Electric-field-induced Schiff-base deprotonation in D85N mutant bacteriorhodopsin. Proc Natl Acad Sci U S A 93: 11618–11621. doi:10.1073/pnas.93.21.11618. PubMed: 8876185. 8876185 10.1073/pnas.93.21.11618 PMC38107

[32] Moltke S, Krebs MP, Mollaaghababa R, Khorana HG, Heyn MP (1995) Intramolecular Charge Transfer in the Bacteriorhodopsin Mutants Asp85–Asn and Asp212–Asn: Effects of pH and Anions. Biophys J 69: 2074-2083. doi:10.1016/S0006-3495(95)80078-0. PubMed: 8580351. 8580351 10.1016/S0006-3495(95)80078-0 PMC1236441

[33] Marinetti T, Subramaniam S, Mogi T, Marti T, Khorana HG (1989) Replacement of aspartic residues 85, 96, 115, or 212 affects the quantum yield and kinetics of proton release and uptake by bacteriorhodopsin. Proc. Nadl. Acad. Sci. USA 86: 529-53. 10.1073/pnas.86.2.529 PMC286505 2536166

[34] Kalaidzidis IV, Kaulen AD (1997) Cl-dependent photovoltage responses of bacteriorhodopsin: comparison of the D85T and D85S mutants and wild-type acid purple form. FEBS Lett 418: 239-242. doi:10.1016/S0014-5793(97)01390-2. PubMed: 9428720. 9428720 10.1016/s0014-5793(97)01390-2

[35] Saeedi P, Moosaabadi JM, Sebtahmadi SS, Behmanesh M, Mehrabadi JF (2012) Site-directed mutagenesis in bacteriorhodopsin mutants and their characterization for bioelectrical and biotechnological equipment. Biotechnol Lett 34: 455–462. doi:10.1007/s10529-011-0731-4. PubMed: 22270563. 22270563 10.1007/s10529-011-0731-4

[36] Ryan MD, Drew J (1994) Foot-and-mouth disease virus 2A oligopeptide mediated cleavage of an artificial polyprotein. EMBO J 13: 928–933. PubMed: 8112307. 8112307 10.1002/j.1460-2075.1994.tb06337.x PMC394894

[37] Szymczak AL, Workman CJ, Wang Y, Vignali KM, Dilioglou S et al. (2004) Correction of multi-gene deficiency in vivo using a single 'self-cleaving' 2A peptide-based retroviral vector. Nat Biotechnol 22: 589-594. doi:10.1038/nbt957. PubMed: 15064769. 15064769 10.1038/nbt957

[38] Boyden ES, Zhang F, Bamberg E, Nagel G, Deisseroth K (2005) Millisecond-timescale, genetically targeted optical control of neural activity. Nat Neurosci 8: 1263–1268. doi:10.1038/nn1525. PubMed: 16116447. 16116447 10.1038/nn1525

[39] Akerboom J, Chen TW, Wardill TJ, Tian L, Marvin JS et al. (2012) Optimization of a GCaMP calcium indicator for neural activity imaging. J Neurosci 32: 13819-13840. doi:10.1523/JNEUROSCI.2601-12.2012. PubMed: 23035093. 23035093 10.1523/JNEUROSCI.2601-12.2012 PMC3482105

[40] Akemann W, Mutoh H, Perron A, Rossier J, Knöpfel T (2010) Imaging brain electric signals with genetically targeted voltage-sensitive fluorescent proteins. Nat Methods 7: 643–649. doi:10.1038/nmeth.1479. PubMed: 20622860. 20622860 10.1038/nmeth.1479

[41] Shamah SM, Lin MZ, Goldberg JL, Estrach S, Sahin M et al. (2001) EphA receptors regulate growth cone dynamics through the novel guanine nucleotide exchange factor ephexin. Cell 105: 233-244. doi:10.1016/S0092-8674(01)00314-2. PubMed: 11336673. 11336673 10.1016/s0092-8674(01)00314-2

